# Current status of national regulations on tanning bed use and workers' protection from solar ultraviolet radiation: results from a global International League of Dermatological Societies (ILDS) questionnaire study

**DOI:** 10.3389/fpubh.2025.1597621

**Published:** 2025-09-16

**Authors:** Cara Bieck, Antje Alberts, Swen Malte John

**Affiliations:** ^1^Department of Dermatology, Environmental Medicine and Health Theory, Institute for Health Research and Education, Osnabrück University, Osnabrück, Germany; ^2^Division of Occupational Medicine, Department of Occupational Medicine, Hazardous Substances and Health Sciences, Statutory Accident Insurance for the Health and Welfare Services, Hamburg, Germany; ^3^Institute for Interdisciplinary Dermatological Prevention and Rehabilitation (iDerm) at Osnabrück University, Osnabrück, Germany; ^4^Lower Saxony Institute of Occupational Dermatology (NIB), Osnabrück, Germany

**Keywords:** cancer, occupation, prevention, regulation, skin, tanning, ultraviolet radiation

## Abstract

**Introduction:**

Exposure to ultraviolet radiation (UVR) is the most important risk factor for the development of skin cancer. Outdoor workers and people who use tanning beds belong to the high-risk groups for developing this disease. The aim of the present study was to gather data about national regulations on the use of tanning beds and worker's protection from solar UVR.

**Methods:**

Member societies of the International League of Dermatological Societies (ILDS) were asked to participate in a survey by using standardized online questionnaires from January 2023 to January 2024.

**Results:**

A total of 100 dermatologists from 66 different countries answered. Responses were pooled if more than one person responded for one country. Occupationally acquired skin cancer by solar UVR can be recognized as an occupational disease in 29 (43.9%) of 66 responding countries. In 29 (43.9%) of 66 responding countries there are legal instruments available aimed at controlling the cosmetic use of tanning beds. In 48 (72.7%) of 66 countries, the responding member society currently participates in any activities on UVR protection and/or use of tanning beds.

**Discussion:**

The results of the presented study indicate a strong need for further action in terms of skin cancer prevention on different levels. This is of high importance due to the fact that skin cancer is a rapidly increasing global public health concern. This issue is particularly applicable to high-risk groups regarding the development of occupational skin cancer, as (a) outdoor workers as well as (b) people from the general population who may have limited awareness or understanding of UVR exposure risks (e.g., through tanning bed use).

## Introduction

Exposure to ultraviolet radiation (UVR) is the main risk factor for the development of skin cancer. UVR is invisible to the human eye and can be divided into three main types, which differ in their wavelength and biological effect: UVA, UVB and UVC. These types of radiation each have different toxic effects on the skin due of their different penetration rates into the epidermis (1). UVA comprises the longest wavelengths of UVR (315–400 nm) and penetrates deeply into the skin, reaching the dermis and can indirectly cause deoxyribonucleic acid (DNA) damage by triggering the generation of reactive oxygen species (1). UVB radiation (280–315 nm) has a shorter wavelength and therefore is more energetic (2). UVB radiation is primarily responsible for sunburn and direct DNA damage, which can lead to skin cancer (1, 3, 4). UVC (100–280 nm) is the most energetic and potentially most dangerous form of UVR. The UVC and portions of the UVB component of solar UVR are absorbed by the earth's atmosphere—especially molecular oxygen and the ozone layer—and do not reach the earth's surface (2). The strength of solar UVR varies depending on geographical location, season and time of day. The highest intensity is reached in the summer months and near the equator (2).

UVA and UVB radiation are of particular importance for the presented study. While UVA radiation is mainly responsible for skin aging and long-term skin damage, UVB radiation is the main cause of sunburns and acute DNA damage leading to skin cancer (4). In the case of natural UVR from the sun, both UVA and UVB rays are relevant, as both types reach the earth's surface and therefore the human skin. Due to the deep penetration of UVA radiation into the skin it can reinforce the carcinogenic effects of UVB, thereby contributing to the development of skin cancer (5). Outdoor workers are at particularly high risk as they are frequently exposed to sunlight in their working environment (6). In 2019, 1.8 billion workers were exposed to UVR at work which corresponds to about 28.4% of the working age population (7).

Another group at elevated risk for skin cancer are people who use tanning beds. UVR, whether from the sun or artificial sources, increases the probability for the development of skin cancer (8). The social and culturally conditioned ideal of tanned skin—which is present in some societies—encourages the use of inadequate or inappropriate protective measures, thereby increasing the risk of sunburn and growth of the skin cancer epidemic (9). Younger people in particular are targeted by advertisements for tanning bed use (8). Artificial UVR in tanning beds is mainly generated by UVA radiation in order to achieve a more rapid and intensive tan as the mechanism of immediate pigment darkening is triggered. The UVB content in sunbeds is generally lower than in natural sunlight (10) even though biological efficacy is considerably higher than that of UVA. The more intense UVR exposure in tanning beds, coupled with repeated use, can lead to an increased risk of skin cancer. Applied doses may be 10 times higher compared to solar UVA.

Skin cancer caused by UVR includes two main categories: keratinocyte carcinomata, more often referred to as non-melanoma skin cancer (NMSC), and malignant melanoma (MM). NMSC spans basal cell carcinoma (BCC) and squamous cell carcinoma (SCC). In the development of skin cancer, early forms of SCC (“*in situ*”) in the form of actinic keratosis (AK) and Bowen's disease must also be taken into account. These are also caused by UVR and have the potential to develop into SCC, which can therefore be associated with considerable morbidity (11). The incidence of BCC and SCC has increased worldwide in recent years, especially among outdoor workers after years of cumulative sunlight exposure (12). The risk especially for the development of BCC is increased by the joint influence of sunlight exposure and the use of tanning beds. It was found that a 60% risk of developing BCC is associated with a history of severe sunburn when combined with the use of tanning beds (13). Solar UVR is mainly associated with the development of NMSC. These skin cancers often arise in subjects with large cumulative UV doses such as those due to repeated UV exposure in outdoor occupational settings. If NMSC is not detected and treated at an early stage, it can lead to severe tissue damage and metastases.

MM, a rarer but more serious and aggressive form of skin cancer, is primarily caused by intense, intermittent UV exposure, which is also relevant in the development of sunburns. The incidence of MM has increased annually, which can be attributed to increased UV exposure through sunbathing and the use of sunbeds (8). In particular, shorter, intermittent but intense UV exposure, as can occur in tanning bed use, has been identified as a significant contributing factor (4). While solar UVR leads to NMSC primarily through prolonged, daily sun exposure, artificial UVR is particularly associated with the development of MM as it generally provides a higher intensity in a shorter time. A causal relationship between MM and intermittent exposure during leisure time, especially during childhood and adolescence, can be assumed (6).

It is therefore important that national regulations are in place to protect people from risk factors and thereby reduce the number of illnesses. Regarding skin cancer as an occupational disease, current estimates by the World Health Organization (WHO) and the International Labor Organization (ILO) assume that almost one in three deaths from NMSC can be attributed to sun exposure in an occupational context. A review of the data on deaths attributable to occupational skin cancer revealed an 88% increase between 2000 and 2019 (7, 14). These estimates emphasize the significance of ongoing research and efforts to prevent occupational exposure to UVR and the burden of NMSC in this context (7). The International League of Dermatological Societies (ILDS) is committed to dermatology and improving skin health at a global level, and is working closely with the WHO on this initiative (15).

Against this background, the aim of the presented study was to determine the current status of the recognition of various types of skin cancer caused by occupational UVR as an occupational disease and the pre-requisites for this in different countries. Furthermore, legal instruments to control the use of tanning beds and the participation in activities on prevention and education about tanning beds were to be identified. The findings shall support further improvement of the acknowledgment and prevention of skin cancer. Also, the results of this study can help to fill gaps in the legal regulation of tanning beds to stop the global skin cancer epidemic.

## Materials and methods

On the 23rd of January 2023, the ILDS disseminated standardized online questionnaires to its member academic societies. The survey was then run until the 29th of January 2024. In case of more than one person responding for one country, the answers were pooled. If multiple answers from one country were contradictory, a plausibility check was conducted by manually cross-checking a sample of responses against the officially reported legal regulation.

## Results

### Characteristics of the participants

A total of 100 dermatologists responded to the online questionnaires representing a wide range of 66 countries. The highest number of dermatologists responded from Italy (n=10), followed by Japan (*n* = 4), and Australia, France, and Spain (*n* = 3, each). Responses were received from two dermatologists in each of the following countries: Austria, Costa Rica, Denmark, Ecuador, Egypt, Kuwait, Mauritius, Netherlands, Nigeria, Palestine, Philippines, Sri Lanka, Taiwan, United Kingdom (UK), United States of America (USA), and Venezuela. For most of the countries, only one dermatologist responded, viz. Belarus, Bolivia, Brazil, Canada, Chile, Colombia, Congo (Democratic Rep), Croatia, Czech Republic, Dominican Republic, El Salvador, Finland, Georgia, Germany, Greece, Guatemala, Honduras, Iceland, India, Indonesia, Kazakhstan, Malaysia, Mali, Malta, Mauritania, Mexico, Morocco, New Zealand, Norway, Pakistan, Poland, Portugal, Romania, Serbia, Singapore, Slovakia, Sweden, Switzerland, Syria, Tanzania, Thailand, Turkey, United Arab Emirates (UAE), Uruguay, and Vietnam.

### Recognition of skin cancer by ultraviolet radiation as occupational disease

**Figure 1:**
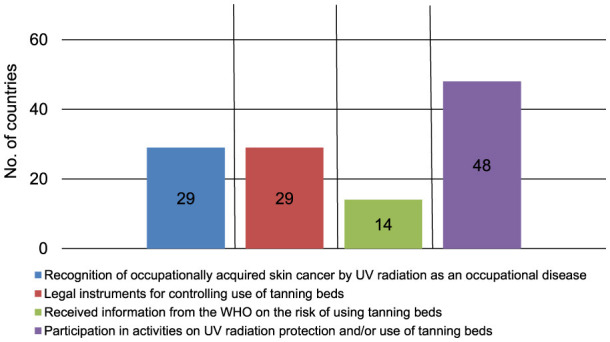
Key findings of the presented survey with 66 individual countries responding; UV, ultraviolet; WHO, World Health Organization.

**Figure 2:**
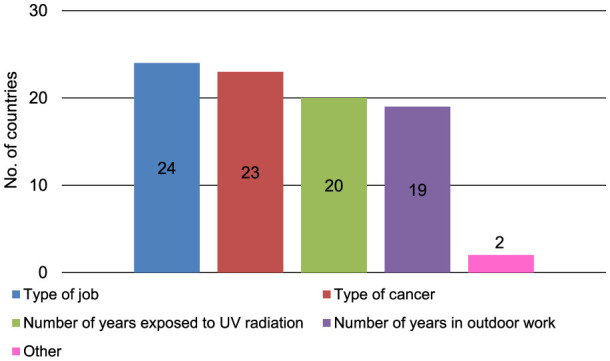
Pre-requisites for recognition of occupationally acquired skin cancer by UVR in 29 (43.9%) of 66 responding countries in which occupationally acquired skin cancer by UVR can be recognized as an occupational disease (multiple answers possible); UVR, ultraviolet radiations.

**Figure 3:**
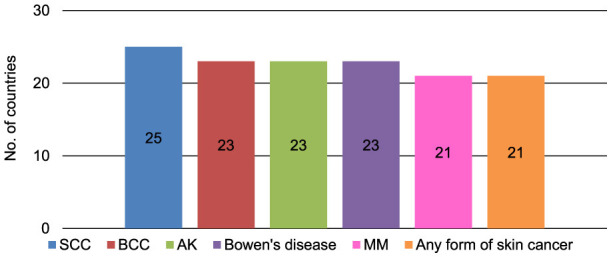
Types of skin cancer acknowledged as occupational skin cancers by UVR in 29 (43.9%) of 66 responding countries in which occupationally acquired skin cancer by UVR can be recognized as an occupational disease (multiple answers possible); AK, actinic keratosis; BCC, basal cell carcinoma; MM, malignant melanoma; SCC, squamous cell carcinoma.

Occupationally acquired skin cancer by UVR can be recognized as an occupational disease in 29 (43.9%) of 66 answering countries (Figure 1). The pre-requisites for recognition in those 29 countries are the type of job in 24 countries (82.8%), the type of cancer in 23 countries (79.3%), the number of years exposed to UVR in 20 countries (69.0%), the number of years in outdoor work in 19 countries (65.5%), and other pre-requisites in two countries (6.9%) for which ‘war veteran from Vietnam and other wars in sun' and ‘not too much leisure time exposure; private exposure versus work exposure, continuous exposure versus intermittent exposure' were mentioned (multiple answers were possible) (Figure 2). Within the 29 answering countries in which occupationally acquired skin cancer by UVR could be recognized as an occupational disease, 25 (86.2%) acknowledge SCC, 23 (79.3%) acknowledge BCC, 23 (79.3%) acknowledge AK, 23 (79.3%) acknowledge Bowen's disease, 21 (72.4%) acknowledge MM, and 21 (72.4%) acknowledge any form of skin cancer as occupational skin cancer by UVR (Figure 3). Two (3.0%) out of 66 answering countries mentioned that although none of the above-mentioned skin cancers are yet recognized as occupational skin cancer, official initiatives are underway to launch an occupational skin cancer national prevention program.

### Legal regulations for the use of tanning beds

Of 66 responding countries, in 29 (43.9%) there are legal instruments available aimed at controlling the cosmetic use of tanning beds (Figure 1). Fourteen (21.2%) of the 66 answering countries have received information from the WHO concerning their work on the risk of using tanning beds (Figure 1).

### Activities on ultraviolet radiation protection and/or use of tanning beds

In 48 (72.7%) of 66 answering countries, the responding member organization currently participates in any activities on UVR protection and/or use of tanning beds (Figure 1). Forty nine (74.2%) of 66 responding countries currently conducting activities on UVR protection and/or use of tanning beds described their activities (Table 1), for which multiple responses were possible.

**Table 1:** Examples of activities on UVR protection and/or use of tanning beds provided by the participants of the survey.

**Activity category**	**Examples of the answers provided by the participants**
Skin cancer campaigns/public relations work	Public lectures by experts, reports in newspapers, annual national skin cancer campaigns, sun awareness weeks, sun awareness months, radio and television campaigns, melanoma day, awareness weeks, routinely provide education about the danger of UVR and encourage people to utilize UV protection through direct campaign and social media platform, sun exposure awareness campaigns in schools and in the general public, national skin-check days
Medical/dermatological actions including patient education	Advancing the diagnosis and medical, surgical and cosmetic treatment of the skin, hair and nails, advocating high standards in clinical practice, education and research in dermatology, supporting and enhancing patient care for a lifetime of healthier skin, hair and nails, equitable sunscreen access, patient education (e.g., on sunscreen use), public education, and education of students and healthcare workers on the harmful effects of UVR, skin checks by dermatologists all over the country free of charge and advice about the danger of UV exposure, patient information and opportunistic training of authorities in municipalities and counties, patient education about sunscreens and sun protection in general, online patient education
Skin cancer research	Research in skin cancer and systemic photoprotection for organ transplant recipients, research in skin cancer/actinic keratosis treatment, skin cancer research, conferences that include reports on dermato-oncology, including issues of prevention and protection from UVR, public lectures by experts and reports in newspapers
Use of tanning beds	Restricting the use of indoor tanning beds, contacting sport schools to ban tanning beds, general ban of tanning beds in some countries
Legislative actions	Cooperation with officials regarding the legislative, skin cancer legislation

## Discussion

The present study shows that in 29 out of the 66 countries, occupational skin cancer caused by UVR is recognized as an occupational disease. The recognition of this disease varies depending on factors such as the type of occupation, the type of cancer, and the duration of UV exposure. Furthermore, a considerable number of these countries engage in initiatives aimed at safeguarding against UVR and regulating the use of tanning beds. Specifically, 43.9% of countries have implemented legal measures to regulate the cosmetic use of tanning beds.

One hundred dermatologists from 66 seven different countries answered. It is notable that European countries are particularly prevalent in the data set (26 countries). With 10 participants, Italy was the country with the highest number of responses. As the ILDS has 217 member societies from 104 different countries, but responses were received from only 66 countries, it can be suggested that there is probably a stronger commitment to UV protection in the responding countries (16).

The results demonstrate that less than half of the responding countries (43.9%) recognize occupationally acquired skin cancer caused by UVR as an occupational disease. This is a problematic situation in terms of global public health, particularly given the elevated risk factor for developing skin cancer, which is especially pertinent in the context of outdoor workers. Furthermore, there is a notable dearth of acknowledgment of NMSC as an occupational disease in the countries where such acknowledgment is theoretically possible. One potential explanation for the absence of NMSC reporting as an occupational disease is the lack of routine documentation of the correlation between the disease and the occupation in question (17). In countries where recognition is a possibility, the primary pre-requisites for acknowledgment are predominantly the type of job (82.8%) and the type of cancer (79.3%). Trakatelli et al. (18) demonstrated a strongly increased risk for AK, BCC and SCC among farmers, construction workers and other outdoor workers compared to indoor workers, especially if they spend many hours outdoors daily and for many years (18). Men and older age groups are particularly affected (7). However, the active application of sun protection measures is frequently impeded by various barriers. These often relate to individual preferences such as comfort and the perceived impracticality of sun protection measures. In addition, sun protective clothing and sun protection products should also be adapted to the specific working conditions (19). As it may be challenging for outdoor workers to avoid the sun at peak times, sun protection measures such as the use of sunscreen should be utilized and encouraged. Therefore, it is necessary to break down the barriers (20). Educational measures that can positively influence the sun protection behavior of outdoor workers can include the provision of information material (e.g., brochures), reminders about sun protection through, for instance, the use of signs or cell phone messages, and specific training on the subject of skin cancer prevention and sun protection (12). An increase in knowledge and an appropriate perception of risk can positively influence sun protection behavior in the workplace (12). In the context of primary prevention, the use of personal protective equipment is of particular relevance. In this, the so-called TOP principle is of high importance, in which measures are to be used in a hierarchical order: First, technical measures (e.g., shading); second, organizational measures (e.g., shifting working hours from solar UVR peak hours); and third, personal measures (e.g., clothing, sunscreen use). The personal measures further may include the use of sunglasses, long clothing and headgear that protects against UVR. In regard to sunscreen, it is essential to ensure that the product provides protection against both UVA and UVB radiation, is waterproof and has a sun protection factor of at least 30 (preferably 50+) (12). Furthermore, primary prevention for occupational skin cancer has been demonstrated to be a cost-effective approach, straightforward to implement and enforceable through legislation at both national and supranational levels (21). It has also been demonstrated that the provision of sunscreen eliminates the barrier to access sunscreen. One potential solution is to store sunscreen in a convenient location, for example on a key fob, to reduce the inhibition threshold for reapplying sunscreen. Moreover, the application of sunscreen appears to be more prevalent among outdoor workers than the use of long clothing in the summer months (20), which is one example for why education on the correct implementation of measures according to the TOP principle is necessary.

In connection with the pre-requisites for recognition, the distinction between occupational and private UV exposure was mentioned, among others. It should be noted that it can be challenging to determine the exact source of exposure when diagnosing occupational skin cancer. It is possible that private exposure, such as sunbathing, may have an additional negative effect on the skin, particularly when combined with an existing occupational exposure. In countries where skin cancer is recognized as a consequence of occupational UV exposure, a decision must be made at the time of diagnosis as to whether private or occupational exposure plays a greater role. Furthermore, it is important to consider that UV exposure is currently increasing in both fields (occupational and private) due to climate change (22, 23). In the light of this complexity, a precise analysis of the patient's occupation and a detailed examination of the skin, including accurate statements from the patient, is essential in order to ensure an accurate diagnosis. With Wittlich's formula a profound estimation of occupational UVR exposure can be achieved (22).

Sun safety campaigns can also help to improve sun protection behavior both at work and in a private context. One example of this is the “Slip, Slop, Slap” campaign, which has been running in Australia since 1981. In 2007 it was updated to “Slip, Slop, Slap, Seek, Slide” and is directed at the general population which does not exclude the application in the occupational environment, but is just as necessary and useful here (24, 25). The meaning behind this slogan is to **slip** on a (long-sleeved) shirt, **slop** on sunscreen and **slap** on a hat, **seek** shade and **slide** on sunglasses, in order to protect the skin and eyes against solar UVR (24, 25). In Germany an educational patient counseling approach for individual sun protection was developed and tested (26). The personalized counseling approach, which allows for the components to be flexibly adapted to the patient's requirements, was well received by the outdoor workers. Furthermore, it was discovered that despite the prevalent endorsement of enhanced sun protection methodologies among specific occupational groups, the majority of sun protection programmes do not consider the distinctive circumstances of those engaged in outdoor work. It is imperative that this be considered when developing enhanced sun protection strategies (25, 26). The aforementioned examples illustrate the efficacy of educational measures in both the general (private) environment and the professional context, demonstrating their capacity to enhance sun protection behavior. It is, therefore, important that such measures continue to be developed, investigated and promoted.

In this investigation, the number of years exposed to UVR is considered by 69.0%, the number of years in outdoor work by 65.5% of the responding countries which recognize skin cancer caused by UVR as an occupational disease. The acknowledgment of different types of skin cancer as occupational diseases varies considerably between the responding countries, with MM being acknowledged in 79.3% of countries and SCC in 86.2%. Obviously, epidemiological evidence for increased risk of outdoor workers compared to the average population exists only for SCC, AK, Bowen's disease and BCC (7, 27–29), it is lacking for melanoma, even though some efforts have been made to establish an association (30). However, the acknowledgment of any form of skin cancer or its precursors, such as AK and Bowen's disease, remains inadequate in numerous countries. For outdoor workers affected by skin cancers that are not yet recognized as work-related, this can have an impact on their treatment options (31, 32). In the absence of such recognition, the affected employees might not be entitled to work-related compensation. This can place them at a disadvantage when it comes to covering the costs of medical treatment. To improve the medical care of outdoor workers with occupational skin cancer an intense education of the authorities and health facilities and standardized reporting of UV-related skin cancer to population-based cancer registries should be provided. In addition, country specific legislation should be adapted to ensure access to health services for all affected outdoor workers. An important Position Statement in this regard, drawn up as a result of the first Multi-Stakeholder Summit on Occupational Skin Cancer (Paris, 2019), set out the following five points to be achieved in this context (17):

1. Policymakers should improve the legislative framework to protect outdoor workers more effectively and build accessibility for regular screenings and thus earlier treatments. In the European Union (EU), NMSC should be officially recognized as an occupational disease within the next legislative period.
2. Doctors, other health professionals and policymakers should work together to ensure standardized EU-wide registration of NMSC.
3. Employers should use tools to monitor exposure levels to UVR in the workplace. They shall also implement cost-effective techniques for sun-safe behavior and ensure regular skin cancer screenings for outdoor workers.
4. Doctors and other health professionals should improve reporting of occupational NMSC (including AK).
5. Patient advocacy groups, doctors and other health professionals as well as employers should collaborate to promote skin cancer prevention and sun-safe working practices and to address the unmet needs of retired outdoor workers with persisting NMSC.

The achievement of these five important goals which are also part of the Global Call to Action (33) could provide a better situation for outdoor workers regarding the prevention, treatment, and acknowledgment of occupational skin cancer.

With regard to the cosmetic use of tanning beds, the results indicate that legal instruments for controlling this are available in < 50% of the responding countries. Mathes et al. (8) compared the legal regulations on tanning bed advertising and information requirements in North America, Australia/New Zealand and Europe. Their findings revealed heterogeneity of 131 legislative units, with 81 (60%) having some form of legal regulation. The authors recommend an international exchange in order to develop global standards on the regulatory framework (8). It is at this juncture that the WHO and ILDS are especially involved. However, the results on receiving information from the WHO concerning their work on the risk of using tanning beds reached only 21.2% of the responding countries which is an unfortunately low number. The reasons for this are unclear and should be documented in order to address this gap in knowledge and improve the available information about the risks associated with tanning beds. In order to reduce the incidence of skin cancer it is necessary to limit the exposure of the population to UVR from tanning beds. It is therefore necessary to educate—especially young—people about the risks associated with UV exposure and the importance of UV protection, given that they represent the primary target for the sunbed industry (8). This is problematic because increased UV exposure at a young age can significantly elevate the risk of developing skin cancer, particularly MM and BCC (6). Furthermore, the use of tanning beds for cosmetic purposes should be strictly regulated by the government, or better, prohibited, which is already the case in some countries (see Table 1), particularly for adolescents and children. This necessitates the implementation of comprehensive educational initiatives and international discourse to facilitate the introduction of suitable measures. It would also be feasible to impose conditions on the sunbed industry, which is currently focusing its advertising primarily on young people. An obligation to provide information concurrently would be a viable option, but this could appear to be challenging to implement due to the involved economic interest. Nevertheless, it is incumbent upon the service provider to ensure that the customer is fully informed, a responsibility that must be defined by legal regulations. Consequently, education at an early age, e. g. in school, is of paramount importance to ensure that young people are aware of sun safety and the risks associated with the use of tanning beds. Also, Suppa et al. (34) investigated the prevalence and determinants of using tanning beds among 30 member countries of Euromelanoma, a European campaign for primary and secondary skin cancer prevention (34). As expected, they found a lower prevalence than in previous reports, as the data came from a campaign for skin cancer screening. Prevalence rates were higher in northern countries with low levels of sunlight, with the exception of Italy and Spain. Age and gender were identified as important determinants. Young adults and women were the most frequent users of tanning beds. The highest prevalence rates of young adults using tanning beds were found in the Baltic countries and among adolescents in Scandinavian countries (34). This emphasizes once again the need for prevention work and legal regulations, particularly in adolescents. In the light of the still culturally and socially recognized aesthetic ideal of tanned skin, it must be questioned whether alternatives to UV exposure are more appropriate for tanning the skin. Newton et al. (35) investigated popular self-tanning products in this regard and found that they often contain contact allergens (e.g. propylene glycol, linalool, fragrances, etc.). Whilst these products may represent an alternative to UVR, they harbor the risk of allergic reactions (35).

Fortunately, the number of countries with current participation in activities on UVR protection and/or against the use of tanning beds, is high (72.7%). Previous multicomponent intervention studies have demonstrated the impact of knowledge and awareness on sun protective behavior in outdoor workers (36, 37). However, greater commitment from employers and regulation by the health and safety authorities is recommended to ensure consistent and effective implementation of protective measures and to promote long-term behavior change that reduces the risks of UVR exposure in the workplace (37). As illustrated in Table 1, the execution of campaigns and public relations initiatives can be implemented very differently. The fact that there are differences between sexes and age groups in the field of increased risk for skin cancer—women and young adults for tanning beds (34), men and older age groups for occupational skin disease (7)—also shows that educational measures are needed for all sexes and age groups to promote general awareness of sun safety and skin protection. It is relevant to once again cite the successful “Slip, Slap, Slop, Seek, Slide” campaign in Australia, which even combines its slogan with a catchy song that calls for people to behave *sun smart* when they are outside, whether at work or in a private context (24). The Australian Cancer Council identified an effective strategy for raising public awareness about sun safety and promoting simple, effective methods for protecting oneself from solar UVR. Due to the SunSmart programme there has been a cultural transformation toward sun protection norms, schools and workplaces have adopted sun protection policies as a result of this initiative (38). It is similarly possible for sun protection campaigns to achieve popularity in other countries if they are disseminated in an appropriate manner. During the launch of “Slip Slop Slap” in 1981, the central distribution medium for videos was television. In the current era, it is feasible to disseminate such initiatives via the Internet, for instance, through the placement of advertisements adjacent to video content, e. g. like it is frequently the case with YouTube. In Germany a campaign has been initiated with a specific focus on skin health in occupational contexts entitled “haut und job” (“skin and job”). The campaign website contains information about occupational skin diseases, prevention, treatment and reporting, as well as download material and links to further resources (39). This campaign is only one part of the European initiative “Healthy Skin @ Work” of the European Academy for Dermatology and Venerology (EADV) with the slogan “Your skin—the most important 2 m^2^ of your life” (40). Another example is the aforementioned Euromelanoma, which was founded in 1999. This organization focuses on the general public through its website and an annual campaign, drawing attention to secondary prevention, screening and treatment options for skin cancer in addition to primary prevention (41). For dermatologists and other health professionals, scientific articles are regularly published and studies are carried out with the help of the member states, e.g., the comparison of tanning bed use in different countries (34, 41). As a third target group, Euromelanoma addresses governments by organizing special events to highlight problems and secure political support (41).

One limitation of this study is the relatively low number of countries that responded to the survey. Given that only 66 of the 104 member countries of the ILDS responded, it would be worth considering how to encourage greater participation from countries in the future. Regarding the manual pooling of cases in which more than one person answered for one country, one limitation is that human error can occur when collating and interpreting the data. Contradictory answers could be subjectively assessed, which might lead to distortions. In addition, the manual process can impair the reproducibility. Although there was a manual plausibility check, it cannot be ruled out that inaccuracies occurred due to subjective judgements. However, it cannot be assumed that the significance of the results is restricted by these limitations, given that possible discrepancies are minimal and unlikely to have a major impact on the findings.

Further research could build on the present findings by linking the questionnaire results with national data on skin cancer incidence and mortality rates. A comprehensive analysis could provide insights into the potential influence of disparities in reporting structures, practices on the documentation and outcomes of occupational skin cancer cases. Furthermore, it would be worthwhile to explore whether regulatory measures, such as the regulation of tanning bed use or the formal recognition of skin cancer as an occupational disease, have an impact on reported skin cancer rates across countries. These directions offer promising opportunities for further investigation and could contribute to a deeper understanding of systemic factors influencing occupational skin cancer reporting and prevention.

## Conclusion

In the light of the results of this study, it is recommended that the recognition of occupational skin cancer as an occupational disease should be reconsidered and implemented in many countries to ensure adequate care for affected outdoor workers. With regard to the various types of skin cancer, the links between outdoor work and types of skin cancer, including the precursors (AK, Bowen's disease), should be disclosed and taken into account. It is recommended that the international exchange that is already taking place at the Multi-Stakeholder Summit on Occupational Skin Cancer should be further promoted in order to make the political authorities aware of the problem and the need for recognition (42–44). In terms of prevention, awareness campaigns should not only target the general population, but should also focus on UV exposure in the workplace and its effects on the skin. The examples given in this study show that there are various effective options here. Training and awareness-raising measures for employees and the cooperation of employers, for instance through the provision of sunscreens, should also be promoted in this regard. It is important to implement sun protection measures, for which reminders can be established as means of ensuring compliance. In addition to primary prevention in outdoor workers, it is necessary to make available to this high-risk group secondary and tertiary prevention, and possible contact points in the case of diagnosed or suspected skin cancer.

In the light of the legal regulations on protection from artificial UVR in tanning beds, stricter guidelines should be implemented, particularly for adolescents and, ideally, also for (young) adults as well. The sunbed industry and advertisers should be subject to requirements that include comprehensive information for customers in addition to advertising. Another potential regulatory measure would be the establishment of guidelines for using tanning beds with regard to frequency, duration and the maximum recommended radiation dose, which should be regularly reviewed and updated. It should also be determined whether alternative methods to achieve a tanned appearance, such as self-tanning lotions, are more appropriate in this context, possible contact allergies should be considered.

In both described scenarios, the implementation of preventive measures to reduce UV exposure and the promotion of increased education and legal regulation are of central importance. In conclusion, the results of the presented study indicate a pressing need for further action in terms of skin cancer prevention at various levels, spanning not only the leisure but especially the occupational sector. This is of significant importance given the rising prevalence of skin cancer as a global public health concern as this disease disproportionately affects high-risk groups, including outdoor workers, and people with a proclivity for unprotected UVR exposure, such as tanning bed use.

## Data Availability

The original contributions presented in the study are included in the article/supplementary material, further inquiries can be directed to the corresponding author.
